# Long-term care needs and hospitalization costs with long-term care insurance: a mixed-sectional study

**DOI:** 10.3389/fpubh.2024.1226884

**Published:** 2024-04-02

**Authors:** Tiantian Che, Jia Li, Jun Li, Xiaobo Chen, Zangyi Liao

**Affiliations:** ^1^School of Public Administration, Dongbei University of Finance and Economics, Dalian, China; ^2^School of Investment Project Management, Dongbei University of Finance and Economics, Dalian, China; ^3^School of Political Science and Public Administration, China University of Political Science and Law, Beijing, China

**Keywords:** long-term care needs, hospitalization costs, long-term care insurance, social hospitalization, long-term care services

## Abstract

**Background:**

With the rapid aging of the population, the health needs of the older adult have increased significantly, resulting in the frequent occurrence of the “social hospitalization” problem, which has led to a rapid increase in hospitalization costs. This study investigates whether the “social hospitalization problem” arising from the long-term care needs can be solved through the implementation of long-term care insurance, thereby improving the overall health of the older adults and controlling the unreasonable increase in hospitalization costs.

**Methods:**

The entropy theory was used as a conceptual model, based on data from the China Health and Retirement Longitudinal Study (CHARLS) in 2015 and 2018. The least-squares method was used to examine the relationship between long-term care needs and hospitalization costs, and the role that long-term care insurance implementation plays in its path of influence.

**Results:**

The results of this study indicated that long-term care needs would increase hospitalization cost, which remained stable after a series of tests, such as replacing the core explanatory variables and introducing fixed effects. Through the intermediary effect test and mediated adjustment effect test, we found the action path of long-term care needs on hospitalization costs. Long-term care needs increases hospitalization costs through more hospitalizations. Long-term care insurance reduces hospitalization costs. Its specific action path makes long-term care insurance reduce hospitalization costs through a negative adjustment of the number of hospitalizations.

**Conclusion:**

To achieve fair and sustainable development of long-term care insurance, the following points should be achieved: First, long-term care insurance should consider the prevention in advance and expand the scope of participation and coverage; Second, long-term care insurance should consider the control in the event and set moderate levels of treatment payments; Third, long-term care insurance should consider post-supervision and explore appropriate payment methods.

## Introduction

In recent years, China’s aging population has entered a rapid development stage. By the end of 2022, the number of older adult people aged 60 and above has reached 280.04 million, accounting for 19.8% of the national population. Health problems among older adults are becoming increasingly prominent, with more than 78% of them suffering from more than one chronic disease, and the number of older adults with disabilities is increasing (1). These factors contribute to increased demand for long-term care. China began exploring the establishment of a long-term care insurance system in 2013. In 2016, 15 cities were selected as pilot sites for the long-term care insurance system, and in 2020, an additional 14 pilot sites were added. Before the implementation of the long-term care insurance pilot, long-term care needs were often met through social hospitalization, leading to excessive growth in hospitalization costs. At the same time, with changes in family structure, the traditional family care function is gradually weakening, and the high fees and service quality of social care institutions make it impossible to effectively meet the long-term care needs of persons with disabilities. The primary purpose of the long-term care insurance fund is to cover the costs incurred by qualified institutions and personnel in providing basic care services. Differentiated treatment and guarantee policies are implemented based on the level of care required and the mode of service provision, with an emphasis on encouraging the use of home and community care services. In November 2021, the “14th Five-Year Plan Proposal” noted the need to “steadily establish a long-term care insurance system”. In his report to the Communist Party of China’s 20th National Congress in October 2022, Xi Jinping underlined the necessity of “establishing a long-term care insurance system”. In this context, whether the long-term care insurance system pilot works requires clarification. Moreover, does this system better address the long-term care needs of persons with disabilities and reduce hospital costs?

The current scholarship is more likely to suggest that the contradiction between the increase of long-term care needs and the insufficient supply of care services has increased demand for nonmedical hospitalization (1), which may lead to the increase of hospitalization costs, but no scholars have proven this point. There is no consensus among the academic community on the role of long-term care insurance’s impact on long-term care needs on hospitalization costs. Many scholars have found that the implementation of long-term care insurance can reduce hospitalization rates and stay duration (2–4), and empirical studies in China found that the implementation of long-term care insurance can effectively reduce medical expenses (5–11); some scholars also found that the implementation of the long-term care insurance system may have increased hospitalization stay duration for patients (12); others have reached different conclusions through empirical studies. By evaluating the effect of the implementation of the long-term care insurance system in Qingdao, some scholars found that the *per capita* cost only showed a short-term decrease and then a continuous increase (13). Some scholars found heterogeneity in the effects of different long-term care payment methods on cost payments, hospitalization costs, etc. (14). After an empirical study, Wang and Feng found that the home care subsidy reduced medical expenses, while the institutional care subsidy did not significantly impact medical expenses (15).

This study asks the following questions. How do long-term care needs impact hospitalization costs? What is the mechanism of this influence? What role does long-term care insurance play? This study provides new empirical evidence to clarify these issues. To achieve this, the study explores the effect of long-term care needs for hospitalization costs based on an entropy theory perspective, and crucially, the important role played by long-term care insurance in the mechanism of its influence is worth exploring. This study provides a new theoretical framework for the study of the relationship between long-term care needs and hospitalization costs. In addition, it also provides theoretical support for the practice of long-term care insurance.

## Theoretical analysis and research hypotheses

The current research is rather fragmented, especially the lack of systematic exploration of the relationship between long-term care needs and hospitalization costs, which limits the development of long-term care insurance practice. To address the above problems and considering the complexity of long-term care service system, this paper applies entropy theory to study the relationship between long-term care needs and hospitalization costs from a game theoretical perspective, so as to enrich the relevant theoretical basis and provide reference for long-term care insurance practice. The concept of entropy, proposed by Clausius, is derived from thermodynamics. It indicates the degree of disorder in a material system; the higher the degree of disorder, the higher the entropy value. An increase in entropy can be derived from the second law of thermodynamics: in isolated systems, the system state always transitions from order to disorder, and the quantity that causes this state transition is called the entropy-increasing factor (16). However, it varies in open systems. Puligotzin introduced the concept of a negative entropy factor: for open systems, the system and the outside world for energy or material exchange will form an entropy flow, increased entropy inside the system transfers to outside the system, or the negative entropy outside the system flows into the system so that the system transforms from disorder to order to achieve a stable equilibrium. With the development of the depth and breadth of entropy theory, it has been widely applied in social science fields.

**Figure 1:**
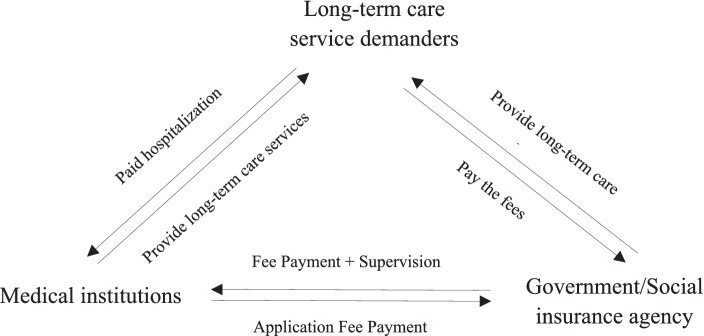
Interaction and relationship of three actors in the long-term care service system.

The factors influencing hospitalization costs involve several dimensions, such as individual characteristics, family structure, social insurance, and health behavior. Although hospitalization costs involves many and mixed interest groups and factors, the aspects that lead to its rapid growth can be summarized as long-term care service demanders, medical institutions, and government/social insurance institutions. All three influence each other and play important roles in the game process, and the specific relationships among the three are shown in Figure 1. The three players will have different game strategies in the game process. First, long-term care demanders want to pay less for better long-term care services, and their game strategy is to choose inpatient or nursing care facilities; second, the government/social insurance agencies are responsible for fund raising, payment and supervision, and their game strategy is to choose the way and intensity of payment; third, medical institutions aim to provide quality services and ensure the sustainable operation of medical institutions, and their game strategy is to provide appropriate or excessive medical services, and their game strategy is to provide appropriate or excessive medical services. Firstly, the system was a closed system prior to the establishment and implementation of the LTC guarantee, and the use of entropy theory as an analytical framework for this study can help us to understand and quantify the informational uncertainty and stochasticity associated with the lack of LTC guarantee. In the current care system, due to the lack of institutional support for long-term care insurance, people in need of long-term care need to purchase long-term care services from medical institutions, which leads to uncertainty and complexity in the flow of information, and is an important entropic factor that leads to an increase in the cost of hospitalization, and the resulting frequent occurrence of the phenomenon of “social hospitalization,” which leads to a sharp increase in hospitalization costs. The resulting high incidence of “social hospitalization” can lead to a sharp increase in hospitalization costs.

Therefore, the following hypothesis is proposed:

*H1*: An increase in long-term care needs leads to increased hospitalization costs.

**Figure 2:**
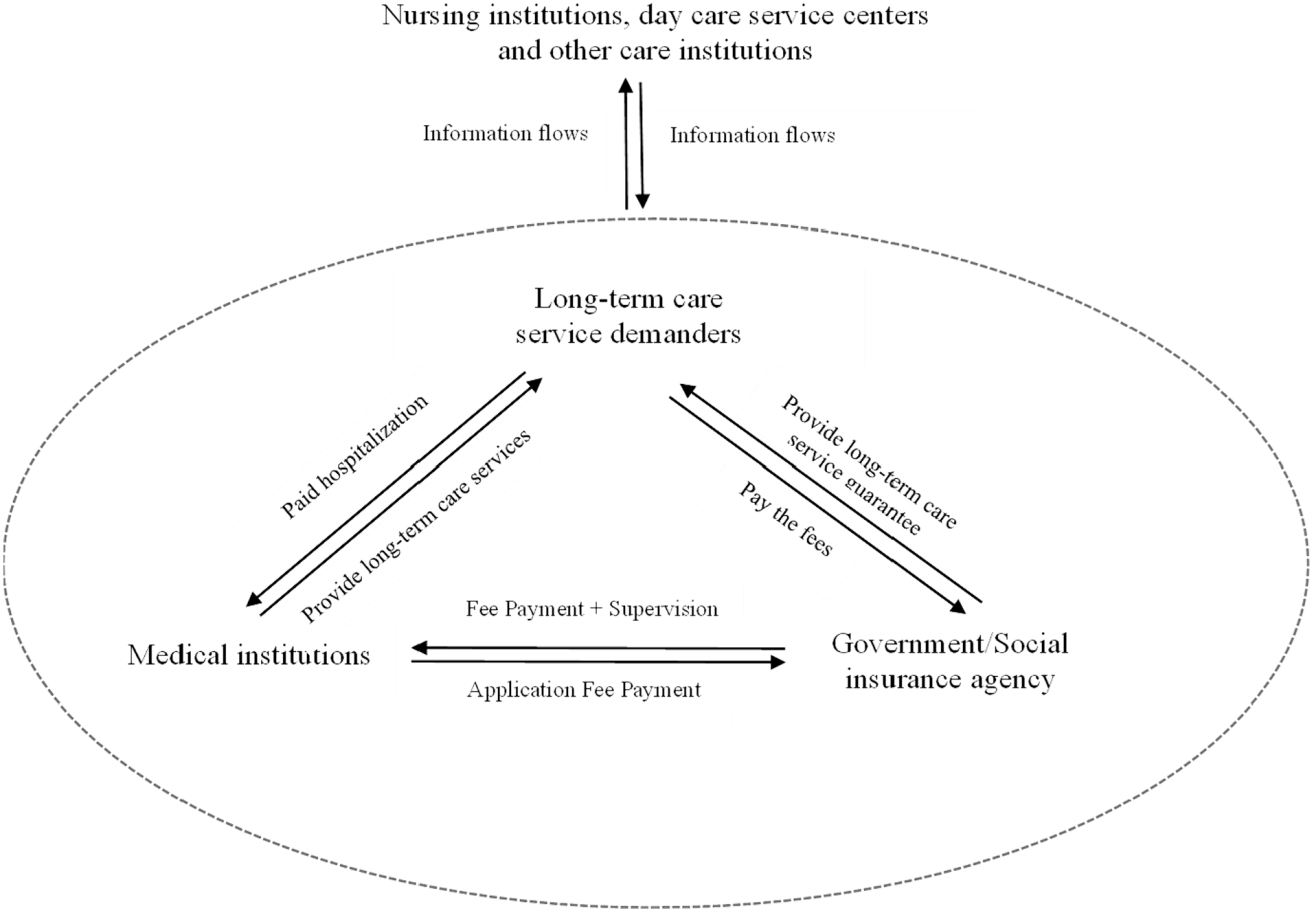
Long-term care delivery systems and the external environment.

Secondly, as pressure on the operation of the Medicare fund increased and the social problem of insufficient long-term care security arose, the long-term care insurance system emerged. With the establishment and implementation of a long-term care insurance system, government policy support and financial subsidies have promoted nursing institutions, day care service centers, and other care institutions, thus forming a long-term care service system beyond medical institutions. The system has become an open system. Entropy theory can be used to optimize decision-making and information flow in order to solve the problem of inadequate long-term care service protection. By analyzing the entropy and uncertainty of information and establishing an information-sharing and exchange platform to promote cooperation and information-sharing between care institutions and medical institutions, government/social insurance institutions and long-term care demanders, bottlenecks and problems in information transfer can be identified and solved, thereby improving the efficiency and quality of long-term care services, as shown in Figure 2. The establishment and implementation of a long-term care insurance system have two effects. On the one hand, it is believed that long-term care insurance raises the income level of its users through subsidies, which indirectly increases demand for “medical care instead of nursing care”, resulting in an entropic effect that increases hospitalization costs. On the other hand, long-term care insurance subsidizes long-term care user costs, which effectively reduces the burden of care for long-term care users and converts their demand for hospitalization into institutional care. This negative entropy effect can, to a certain extent, alleviate the sharp increase in hospitalization costs caused by the “social hospitalization” problem.

Consequently, the total entropy change in an open long-term care service system can be expressed by the following equation:


$$dS=d_{i}S+d_{e}S$$

In equation (1), medical demand for long-term care is an irreversible entropy increase within the long-term care service system, constant with $d_{i}S>0$ ; $d_{e}S$ this indicates that the long-term care needs is met by the care institutions after the long-term care insurance was established, and the long-term care service system receives a positive or negative entropy flow from the outside world. The long-term care insurance system can transform the medical needs of some long-term care users from “medical care instead of nursing care” into nursing care needs, at which time the external environment flows into the long-term care delivery system with negative entropy, which can curb the sharp increase in hospitalization costs. Thus, we propose the following hypotheses:

*H2*: Long-term care insurance can negatively moderate the relationship between long-term care needs and hospitalization costs.

*H3*: The interaction effect of long-term care needs and the long-term care insurance system impacts hospitalization costs through the mediating effect of the number of hospitalizations.

## Data sources, variable selection, and model setting

### Data sources and processing

The China Health and Aging Tracking Survey (CHARLS) 2015 and 2018 data were used for this study because, which were obtained in 2015 and 2018 respectively, after combining them with the PSU file, it was discovered that they contained data for 12 cities from the initial set of pilot cities. As Qingdao did not meet the criteria for double differencing because the pilot long-term care insurance system was not implemented until 2012, the final 11 pilot cities chosen for this study were Chengde, Qiqihar, Ningbo Shanghai, Shangrao, Jingmen, Chongqing, Anqing, Guangzhou, Chengdu, and Suzhou.

## Variable settings

### Explained variable

Hospitalization costs served as an explanatory variable. The question “What is the approximate total cost of hospitalization in the past year?” is the source of this variable. The total cost of hospitalization in this study was logarithmic to account for non-normality in the study methodology.

### Explanatory variables

The need for long-term care was used as an explanatory variable. Since long-term care needs statistics are scarce, and long-term care insurance in China is still in the pilot stage, proxy variables are used to represent long-term care insurance demand in China. Three factors were chosen to gage the need for long-term care based on the applicability and dependability of the data sources: natural demand (amount of disability, number of chronic illnesses, and difficulty of living), objective demand (residence pattern), and alternative demand (supplementary and commercial medical insurance). In this study, the weights of each indicator were calculated using the entropy weighting method, which is based on the degree of variation of each indicator to determine the indicator. The specific process of the entropy method is described in the Appendix. It is reasonable to use the entropy weighting method to calculate long-term care needs because it allows a more objective representation of long-term care needs.

### Control variables

In order to reduce the influence of self-bias on the research results, this study incorporated a series of control variables. The control variables in this study contained the following aspects: (1) personal characteristics: age, sex, marriage, education level, household registration, and retirement; (2) household characteristics such as household income, number of children, and living with children; (3) social insurance characteristics, such as social health insurance and social pension insurance; and (4) health behaviors: smoking, alcohol consumption, self-medication, and medical satisfaction.

### Moderating variable

In this study, long-term care insurance served as the moderating factor. Since the long-term care insurance pilot was conducted after 2015 for the treatment group, 2015 was the pre-treatment (*t* = 0) and 2018 was the post-treatment (*t* = 1). The treatment group (treat) had a dummy variable before and after the introduction of long-term care insurance (*t*). Table 1 presents variable definitions and descriptive statistics.

**Table 1:** Variable definitions and descriptive statistics.

Variable type	Variable name	Definition	Average value	Standard deviation
Explained variables	Hospitalization costs	Hospitalization costs in the past year (in dollars)	0.174	1.264
Explanatory variables	Long-term care needs	Long-term care needs composite index	0.015	0.038
Adjustment variables	Long-term care insurance	treat and t the cross term of	0.213	0.409
Intermediate variables	Number of hospitalizations	Number of hospitalizations after taking the logarithm, in days	1.006	0.684
Personal characteristics	Age	Continuous variables of population age in years	60.507	10.587
	Sex	Female = 1; Male = 0	0.498	0.500
	Marriage	Married = 1; No = 0	0.811	0.391
	Education level	Elementary school and below = 0, middle school = 1; high school and junior college = 2; College, bachelor’s degree and above = 3	0.436	0.700
	Household Registration	Urban = 1; Rural = 0	0.525	0.499
	Retirement	Retirement = 1; No = 0	0.148	0.355
Family characteristics	Household income	Annual household income after taking the logarithm (in dollars)	7.638	3.749
	Number of children	The continuous variable of the number of children in years	2.713	1.724
	Children living together	Living with children = 1; No = 0	0.295	0.456
Social insurance features	Social health insurance	Participate in social health insurance = 1; No = 0	0.976	0.155
	Social pension insurance	Participate in social pension insurance = 1; No = 0	0.657	0.475
Health behavior features	Smoking	Smoking = 1; No = 0	0.278	0.448
	Drinking	Drink = 1; No = 0	0.306	0.461
	Self-treatment	Self-treatment = 1; No = 0	0.505	0.500
	Medical satisfaction	Satisfactory = 0; fair = 1; poor = 2	0.784	0.697

### Mediating variable

The number of hospitalizations was the mediating variable. The enquiry “How many times have you been hospitalized in the past year?” led to a variable number of hospitalizations. The number of days was treated logarithmically to account for non-normality in the research procedure.

## Model setting

### Baseline regression

The least squares method was employed to determine whether long-term care should result in higher inpatient expenses. The baseline regression was built as follows to evaluate Hypothesis 1.


(1) $$HS=\alpha_{0}+\alpha_{1}LN+\alpha_{2}X+\mu$$

HS Represents hospitalization costs; LN represents long-term care needs; and X represents the control variables, and $\alpha_{0}$ , $\alpha_{1}$ , and $\alpha_{2}$ are the parameters to be estimated; and μ denotes the random error term.

### Mediating effect tests

To test Hypothesis 3, the number of hospitalizations was introduced as a mediating variable HN . The mediating effect model is developed as follows:


(2) $$HN=\beta_{0}+\beta_{1}LN+\beta_{2}X+\varepsilon_{1}$$

(3) $$HS=\gamma_{0}+\gamma_{1}LN+\gamma_{2}HN+\gamma_{3}X+\varepsilon_{2}$$

In the above formula, HN is the mediating variable number of hospitalizations, and X are the control variables, the $\beta_{0},\;\beta_{1},\;\beta_{2},\;\gamma_{0},\;\gamma_{1},\;\gamma_{2}\text{,}$ and $\gamma_{3}$ are the parameters to be estimated, and $\varepsilon_{1},\;\varepsilon_{2}$ are the random error terms. Equation (3) places both long-term care needs and the number of hospitalizations on the right-hand side of the model to study their influence on hospitalization costs. Equation (2) examines the impact of demand for long-term care on the number of hospitalizations. The following factors must be met to determine whether the mediating impact is substantial, according to scholastic test of mediating effect (17): First, the overall impact of the equation’s long-term care needs effect on hospitalization costs (1) α 1 The first criterion is whether there is a considerable overall impact of long-term care needs on hospitalization costs in Equation (1); Second, the impact of long-term care needs on the number of hospital admissions in Equation (2) β 1 The third criterion concerns the significance of the relationship between the number of hospital days and hospital cost in Equation (3). Whether long-term care needs have a considerable impact on the number of hospitalizations in Equations (2, 3), and whether the impact of hospital stay duration on hospitalization costs in Equation (3).

Thus, a mediating moderating impact exists. The addition of moderating variables (LTCI), the cross term of the independent variable and the moderating variable ( $I_{1}$ ), and the cross term of the mediating variable and ( $I_{2}$ ); and the testing of research hypotheses 2 and 3. Following are the exact test procedures to determine whether the long-term care insurance system’s installation has a moderating effect, which is based on research by Ye and Wen (18). The moderating effect is first tested for, then the moderating effect with mediation is tested for, and finally, the moderating effect is tested to determine if it is entirely or partially mediated. As a result, the following equation establishes a model for the moderating effect of mediation:


(4) $$HS=c_{0}+c_{1}LN+c_{2}\mathrm{LTCI}+c_{3}I_{1}+c_{4}X+e_{1}$$

(5) $$HN=a_{0}+a_{1}LN+a_{2}\mathrm{LTCI}+a_{3}I_{1}+a_{4}X+e_{2}$$

(6) $$HS=c_{0}^{\prime }+c_{1}^{\prime }LN+c_{2}^{\prime }\mathrm{LTCI}+c_{3}^{\prime }I_{1}+b_{1}HN+b_{2}I_{2}+c_{4}^{\prime }X+e_{3}$$

In order to reduce the influence of self-bias on the research results, this study employed the technique of difference-in-differences (DID) to control potential confounding factors and biases to some extent. In the above formula, HS represents the cost of hospitalization, LN represents long-term care needs, and HN represents the number of hospitalizations, and LTCI Long-term care insurance is the year ( t ) and whether long-term care insurance is in place ( treat ) interaction term, where LTCI=1 denotes the treatment group after implementation of long-term care insurance and 0 in other cases. $I_{1}$ is the need for long-term care LN and long-term care insurance LTCI and $I_{2}$ is the number of hospitalizations HN and long-term care insurance LTCI is the interaction between the number of hospitalizations and long-term care insurance.

$c_{0}$ , $c_{1}$ , $c_{2}$ , $c_{3}$ , $c_{4}$ , $a_{0}$ , $a_{1}$ , $a_{2}$ , $a_{3}$ , $a_{4}$ , $c_{0}^{\prime }$ , $c_{1}^{\prime }$ , $c_{2}^{\prime }$ , $c_{3}^{\prime }$ , $b_{1}$ , $b_{2}$ , and $c_{4}^{\prime }$ are the parameters to be estimated. Finally, $e_{1}$ , $e_{2}$ , and $e_{3}$ denote the follow-on error terms.

## Results

### Baseline regression

In this study, we analyze the impact of long-term care needs on hospitalization fares after adding a series of control variables. The regression results in columns (1)–(4) of Table 2 after adding personal characteristics, family characteristics, social insurance characteristics, and health behavioral characteristics in that order show that long-term care needs significantly increase hospitalization costs at a statistical level of 5%.

**Table 2:** Baseline regression results.

Variables			OLS model			
			(1)	(2)	(3)	(4)
Independent variable		Long-term care needs	1.560^**^ (0.534)	1.589^**^ (0.537)	1.651^**^ (0.537)	1.214^**^ (0.538)
Control variables	Personal features	Age	0.006^**^ (0.002)	0.006^**^ (0.002)	0.006^**^ (0.002)	0.005^**^ (0.002)
		Sex	0.077^*^ (0.041)	0.078^*^ (0.041)	0.070^*^ (0.041)	0.072^*^ (0.042)
		Marriage	−0.013 (0.055)	−0.005 (0.055)	−0.019 (0.054)	0.000 (0.055)
		*Level of education (ref: primary and below)*				
		Junior high school	0.085 (0.053)	0.092^*^ (0.053)	0.090^*^ (0.052)	0.083 (0.054)
		High school and technical secondary school	−0.018 (0.075)	−0.017 (0.075)	−0.004 (0.074)	−0.025 (0.077)
		College degree, bachelor’s degree or above	−0.121 (0.197)	−0.127 (0.196)	−0.114 (0.197)	−0.110 (0.225)
		Household Registration	−0.017 (0.042)	−0.007 (0.043)	−0.013 (0.042)	−0.026 (0.043)
		Retirement	−0.073 (0.060)	−0.103^*^ (0.061)	−0.096 (0.060)	−0.063 (0.061)
	Family features	Household income		−0.009^*^ (0.006)	−0.010^*^ (0.006)	−0.011^**^ (0.006)
		Number of children		−0.020 (0.012)	−0.024^*^ (0.012)	−0.030^**^ (0.012)
		Children living together		0.112^**^ (0.046)	0.104^**^ (0.046)	0.102^**^ (0.047)
	Social insurance features	Social health insurance			0.195 (0.130)	0.166 (0.128)
		Social endowment insurance			−0.063 (0.044)	−0.013 (0.045)
	Health behavior characteristics	Smoking				−0.061 (0.050)
		Drink alcohol				−0.031 (0.049)
		Self-treatment				0.072^*^ (0.042)
		*Medical satisfaction (reference: satisfaction)*				
		General				0.092^**^ (0.045)
		Not satisfied				0.100 (0.063)
*R*-squared			0.008	0.012	0.014	0.015
Sample size			3,555	3,457	3,420	2,976

***, **, and * denote significance at the 1, 5, and 10% statistical levels, respectively, with standard errors of the robustness of coefficients in parentheses.

### Control variables analysis

The estimation results of one of the control variables are of interest, as exemplified in Column (4) of Table 2. In terms of individual characteristics, the age variable is significantly positive and significant at the 5% statistical level, meaning that the cost of hospitalization increases progressively with age. The gender variable was significantly positive and significant at the 10% level, i.e., the cost of hospitalization was higher for women. In terms of household characteristics, the household income variable is significantly negative and is statistically significant at the 5% level, i.e., as household income increases, the cost of hospitalization decreases. The living with children variable was significantly positive and is significant at the 5% level. In terms of health behavioral characteristics, the self-treatment variable was significantly positive and statistically significant at the 10% level, i.e., the cost of hospitalization increased for those who self-treated. In terms of medical satisfaction, the medical satisfaction variable was significantly negative and significant at the 5% statistical level.

## Robustness tests

### Substitution of explanatory variables

Table 3 presents the results of the robustness test estimates of the impact of long-term care needs on hospitalization costs of replacing the core explanatory variables. Through effective replacement of the core independent variable. The “degree of disability” is used to explore its impact on hospitalization costs, and the degree of incapacity measured separately is an important measure of the ability to look after oneself and can effectively reflect the long-term care needs of an incapacitated person. Therefore, the choice of incapacity degree as a replacement variable for long-term care needs is somewhat justified. Substitution of the core independent variables shows that, with the sequential inclusion of a range of control variables, the degree of disability substantially increases hospitalization costs at the 5% statistical level. These results were generally consistent with the baseline regression results, suggesting that the finding that long-term care needs significantly increased hospitalization costs was strongly robust.

**Table 3:** Robustness tests-replacement of explanatory variables.

Variables	OLS model			
	(1)	(2)	(3)	(4)
Degree of incapacity	0.328^***^ (0.076)	0.283^***^ (0.076)	0.298^***^ (0.075)	0.236^**^ (0.078)
Control variables	Controlled	Controlled	Controlled	Controlled
*R*-squared	0.013	0.016	0.020	0.020
Sample size	2,335	2,266	2,240	1964

***, **, and * denote significance at the 1, 5, and 10% statistical levels, respectively, with standard errors of the robustness of coefficients in parentheses.

### Introduction of fixed effects

Table 4 presents the results of the robustness test estimates of the impact of long-term care needs on hospitalization costs for the introduction of fixed effects. The introduction of time and city fixed effects to explore the impact of long-term care needs on hospitalization costs can effectively control the effects of time and city on the explanatory variables; therefore, it is reasonable to explore the effects of long-term care needs on hospitalization costs, controlling for time and municipal fixed effects, controlling for time and city fixed effects, and adding a range of control variables, long-term care needs still significantly increased hospitalization costs at the 10% statistical level, which was broadly consistent with the previous regression results, suggesting that the result that long-term care needs significantly increased hospitalization costs was strongly robust.

**Table 4:** Robustness tests–fixed effects tests.

Variables	OLS model			
	(1)	(2)	(3)	(4)
Long-term care needs	1.597^**^ (0.648)	1.595^**^ (0.665)	1.655^**^ (0.671)	1.206^*^ (0.642)
Control variables	Controlled	Controlled	Controlled	Controlled
Time fixed effects	Yes	Yes	Yes	Yes
Urban fixed effects	Yes	Yes	Yes	Yes
*R*-squared	0.027	0.030	0.030	0.034
Sample size	3,555	3,457	3,420	2,976

***, **, and * denote significance at the 1, 5, and 10% statistical levels, respectively, with standard errors of the robustness of coefficients in parentheses.

## Impact analysis mechanism

### Mediating effect

To further explore the path of the effect of long-term care needs on hospitalization costs, this study used stepwise regression to test whether the number of hospitalizations had a mediating effect. Table 5 presents the regression results of Equations (1)–(3). As shown in Table 5, when the regression in Equation (2) is performed with long-term care needs as the explanatory variable and the number of hospitalizations as the explained variable, long-term care needs significantly increased the number of hospitalizations. When the number of hospitalizations was used as the explanatory variable for the regression in Equation (3), the number of hospitalizations had a significant positive impact on hospitalization costs. Combined with the regression results of Equations (1)–(3), it was concluded that the path of “long-term care needs-hospitalization duration-hospitalization costs” existed, the number of hospitalizations had a partial mediating effect, and research hypothesis 3 was partially verified.

**Table 5:** Mediation moderating effect test.

Variables	(1) Hospitalization costs	(2) Number of Hospitalizations	(3) Hospitalization costs
Long-term care needs	1.214^**^ (0.538)	1.274^***^ (0.158)	−0.126 (0.518)
Number of hospitalizations			1.052^***^ (0.060)
Control variables	Controlled	Controlled	Controlled
*R*-squared	0.015	0.045	0.109
Sample size	2,976	2,978	2,975

***, **, and * are significant at the 1, 5, and 10% statistical levels, respectively, and the standard error of coefficient robustness is in parentheses.

## Mediating moderating effect

### Direct moderating effect

To clarify the moderating effect of long-term care insurance on the impact of long-term care needs on hospitalization costs, this study incorporated the interaction term of long-term care needs and long-term care insurance into the system for moderating effect analysis. As shown in Column (1) of Table 5, after introducing the moderating variable long-term care insurance and the interaction term of long-term care insurance and long-term care needs, the effect of long-term care needs on hospitalization costs is still significantly positive at the statistical level of 5%, the coefficient of the moderating variable long-term care insurance is significantly negative at the statistical level of 5%, and the coefficient of the interaction term of long-term care insurance and long-term care needs is significantly negative at the statistical level of 10%. It can be seen from the above that long-term care insurance inhibits the positive effect of long-term care needs on hospitalization costs, that is, long-term care insurance has a significant negative moderating effect on the impact of long-term care needs on hospitalization costs.

### Mediated moderating effect

According to Ye and Wen (18), the concrete steps of the research are as follows: First, test the regression coefficient c _ 3 in Equation (4). From Column (1) of Table 6, it can be seen that the coefficient of the interaction term between long-term care needs and long-term care insurance was significantly negative and significant at the 5% level. Thus, long-term care insurance had a negative moderating effect. Second, we tested the regression coefficients in Equations (5, 6). From Column (2) of Table 5, we can see that the coefficient of long-term care needs was significantly positive at the statistical level of 5%. From Column (3), it can be seen that the coefficient of the interaction term between long-term care insurance and hospitalization times was significantly negative at the 1% level. Based on this, long-term care insurance indirectly regulated the effect of long-term care needs on hospitalization costs by adjusting the effect of hospitalization days on hospitalization costs. Third, we tested the regression coefficient in Equation (6). The interaction between long-term care needs and insurance had no significant effect on hospitalization costs. Therefore, the moderating effect of long-term care insurance was fully mediated.

**Table 6:** Mediated moderating effect test.

Variables	(1) Hospitalization costs	(2) Number of hospitalizations	(3) Hospitalization costs
Long-term care needs	2.002^**^ (0.662)	1.229^**^ (0.194)	0.177 (0.624)
Long-term care insurance	−0.149^**^ (0.055)	−0.003 (0.016)	0.019 (0.054)
Long-term care needs * Long-term care insurance	−2.139^*^ (1.106)	0.128 (0.325)	−0.143 (1.051)
Hospitalization times			1.408^***^ (0.067)
Long-term care insurance * hospitalization times			−1.437^***^ (0.135)
Control variables	Controlled	Controlled	Controlled
R square	0.027	0.030	0.030
Sample size	3,555	3,457	3,420

***, **, and * are significant at the 1, 5, and 10% statistical levels, respectively, and the standard error of coefficient robustness is in parentheses.

## Discussion

The baseline regression results show that long-term care needs significantly increase hospitalization costs. In the hierarchy of needs theory proposed by American psychologist Maslow, needs are classified into physiological needs, security needs, social needs, respect needs, and self-actualization needs. Corresponding to this disability, there are different levels of long-term care needs, with higher levels pursued only when the lower ones are met. The physical needs of people with disabilities are mainly in the personal care in long-term care needs, and the safety needs of people with disabilities are mainly in the area of timely, adequate, and effective medical care for long-term care needs. When a person with disabilities is in good health, they have a low level of life care needs and relatively simple medical care needs. These long-term care needs can be met by the person with a disability, by their family, or by a briefer hospital stay. However, when the long-term care needs of people with disabilities rise, the inability of people with disabilities themselves and their families to meet their long-term care needs and the need for specialist medical care leads to the “social hospitalization” problem, where people with disabilities pay for specialist medical care through hospitalization, leading to increased hospitalization costs.

The estimation results of one of the control variables are of interest, as exemplified in Column (4) of Table 2. In terms of individual characteristics, the cost of hospitalization increases progressively with age. Possible reasons for this are that as people begin to decline in physical function as they age, they are exposed to an increased risk of illness, requiring life-saving medical treatment and specialist medical care, leading to increased hospitalization costs. The cost of hospitalization for women are higher than males. Possible reasons for this are that there is a significant sex division of labor in family caregiving, with women taking on more caregiving responsibilities when men need family care, but when women need care, the cost of hospitalization is likely to be lower than the cost of care for men, so women are more likely to undertake hospitalization and have higher hospitalization costs than men, and the effects of education level, household registration, and retirement variables on hospitalization costs are not significant.

In terms of household characteristics, as household income increases, the cost of hospitalization decreases. Possible reasons for this are that as household income increases, the person with disabilities and their family members can choose more options to meet their care needs, with home and institutional care having a substitution effect on inpatient care, leading to a reduction in hospitalization costs. As the number of children increases, hospitalization costs decrease. Possible reasons for this are that as the number of children increases, the children of the person with disabilities can assume more family care responsibilities, meet the care needs of the person with disabilities, and reduce hospitalization costs. The living with children variable was significantly positive A possible reason for this is that as family structure changes, family nucleation increases, living with children decreases, and when the need to care for grandchildren or one’s poor health requires care, one chooses to live with children. However, the nuclear family is often unable to provide long-term home care, and this need for home care translates into a need for medical care, leading to increased hospitalization costs.

In terms of health behavioral characteristics, the cost of hospitalization increased for those who self-treated. Possible reasons for this are that persons who self-treat may be in worse health compared to those who do not self-treat and have relatively more medical care needs, leading to increased costs for their hospitalization. In terms of medical satisfaction, there is a negative correlation between medical satisfaction and the cost of hospitalization. A possible reason for this is that the satisfaction of those who have received medical care is mainly based on the quality, cost, and convenience of medical care, and their medical satisfaction is higher when their health improves after receiving medical care, when the cost of medical care is small and when it is convenient, and those whose health does not improve significantly and whose cost of medical care is higher tend to be in poorer health and have more medical care needs which lead to higher hospitalization costs.

The long-term care insurance inhibits the positive effect of long-term care needs on hospitalization costs. A possible reason is that when people with disabilities pursue lower levels of physiological and safety needs, families and hospitals can meet them, but when the lower levels of physiological and safety needs are met, a person with disabilities will pursue higher levels of needs, including social needs, respect needs, and self-actualization needs. First, in long-term care, social needs are mainly manifested in the emotional and belonging needs of persons with disabilities, but this demand is gradually not met due to changes in the family structure. The implementation of long-term care insurance provides cash subsidies for family members who are responsible for family care, and family members provide care services at home, which can effectively meet the social and emotional needs of people with disabilities and effectively reduce hospitalization costs. Second, concerning long-term care needs, respect needs are manifested in the fact that people with disabilities still need to be respected and understood by their families and society after losing their self-care ability. Third, in terms of long-term care needs, the need for self-realization is manifested in the fact that people with disabilities also need to achieve self-worth by participating in social activities. Long-term care insurance provides home care through subsidies and enables people with disabilities to stay in professional care institutions, providing service guarantees for older adults with disabilities, so that they can choose to receive family or institutional care services and freely choose and enjoy better care services. It can effectively meet their higher level of care needs, reduce “social hospitalization”, and reduce hospitalization costs.

The care services provided by long-term care insurance for families and professional institutions met the long-term care needs of people with disabilities, replacing some of the original medical needs and thereby reducing hospitalization costs. From the mediating effect test and mediating moderating effect test, we can determine the possible influence path of the substitution effect of long-term care insurance. On the one hand, long-term care insurance mobilizes the enthusiasm of families, communities, and professional care institutions by providing financial compensation using a “combination of medical and health care” model. When formal or informal care for people with disabilities is provided by family members or professional caregivers, the social and respect needs of persons with disabilities be met to a greater extent, and their physical and mental health will also be improved to a certain extent, reducing the need for non-medical hospitalization and effectively reducing hospitalization numbers, thereby reducing the cost of hospitalization. On the other hand, people with disabilities who receive care in hospitals before the establishment of long-term care insurance can freely choose to receive care services at home or professional care institutions under the promotion of long-term care insurance, rather than spending a lot of time and economic costs to become “bedridden patients”, effectively reducing the number of hospitalizations and medical expenses.

## Conclusions and policy recommendations

In order to explore the impact of long-term care needs on hospitalization costs, especially the important role of long-term care insurance in its impact mechanism, based on data from the China Health and Retirement Longitudinal Study (CHARLS) in 2015 and 2018, this study used the least squares method to investigate the impact of long-term care needs on hospitalization costs and its path from the perspective of entropy theory. The results showed that long-term care needs to increase hospitalization costs, and this result remained robust after a series of tests, such as replacing core explanatory variables and introducing fixed effects. Through the mediating effect test and mediating moderating effect test, we found the path of the impact of long-term care needs on hospitalization costs. Long-term care needs increased hospitalization costs by positively affecting the number of hospitalizations, and long-term care insurance reduces hospitalization costs. The specific path of action makes long-term care insurance reduce hospitalization costs by negatively regulating the number of hospitalizations. Based on the above conclusions, this study proposes the following policy recommendations:

First, long-term care insurance should consider pre-prevention and expand the scope of insurance and protection. According to this study, long-term care needs is an important factor leading to an increase in hospitalization costs. Expanding the scope of long-term care insurance is important for prevention. From the focus on medical care cost protection to solving basic nursing security needs, the scope of insurance and security has greatly expanded. Families and professional nursing institutions can meet the basic nursing needs of persons with mild disabilities and reduce hospitalization costs; second, long-term care insurance should consider the matter under control and develop appropriate levels of treatment payments. In conclusion, it is found that the payment of long-term care insurance benefits can effectively reduce the economic burden on families, promote the choice of home care methods, replace non-hospitalized medical needs, reduce the number of hospitalizations, and inhibit hospitalization costs. Therefore, in the design of long-term care insurance benefits, differentiated treatment protection policies should be implemented and priority should be given to subsidizing home care services, reducing hospitalization needs, and controlling hospitalization costs. Third, long-term care insurance should consider post-supervision and explore the appropriate treatment payment methods. The payment method for long-term care insurance affects the service choices of people with disabilities. Cash payments may reduce the economic burden of people with disabilities and their families, generate income effects, and release hospitalization needs. In addition, a single cash payment method cannot make timely and effective judgments regarding the health status of people with disabilities. The combination of cash payments and nursing services can effectively monitor the health status of people with disabilities, but also improve the health status of people with disabilities through professional nursing services, reduce medical care needs, and control hospitalization costs.

## Limitations and outlook

Due to the current stage of development of China’s long-term care insurance system, which is in the pilot phase, and the fact that the CHARLS data is only updated until 2018, it is not possible to conduct a comprehensive and up-to-date assessment of the effectiveness of China’s long-term care insurance system. In the future, the authors will consider conducting their own survey based on the implementation of the Chinese long-term care insurance system to facilitate the evaluation of the latest system outcomes.

## Data availability statement

Publicly available datasets were analyzed in this study. This data can be found at: https://charls.charlsdata.com/pages/data/111/zh-cn.html.

## Ethics statement

The questionnaire was provided by the National Development Research Institute at Peking University. The questionnaire employs data anonymization to ensure the confidentiality of enrollees.

## Author contributions

JiL and TC contributed to the study design. TC, JuL, and XC collected and collated the data. TC, JuL, and XC contributed to conceptualization, writing – original draft, writing – review and editing. JiL, ZL, and TC supervised the study and contributed to the writing – review and editing. TC responded to and revised comments from reviewers and editors. The authors read and approved the final manuscript. All authors contributed to the article and approved the submitted version.

## Appendix

The procedure for entropy weighting method is as follows:

Step 1: Identify indicators.

Given n samples and m indicators, let $X_{ij}$ represent the value of the j th indicator for the i th sample ( i = 1, 2, .., n ; j = 1, 2, .., m ).

Step 2: Normalization of indicators.

Normalize the indicators to eliminate scale differences. In this study, all the indicators are positive indicators.

$$X_{ij}=\frac{X_{ij}-\min\left\{X_{1j},\ldots ,X_{nj}\right\}}{\max\left\{X_{1j},\ldots ,X_{nj}\right\}-\min\left\{X_{1,\ldots ,j}X_{nij}\right\}}$$

Step 3: Calculate the proportion of the i th sample value to the j th indicator, and assess the variability of the indicator.

$P_{ij}=\frac{X_{ij}}{\sum_{i=1}^{n}X_{ij}},i$ =1, 2, …, n ; j =1, 2, …, m.

Step 4: Calculate the entropy value for the j th indicator.

$e_{j}=-k\overset{n}{\underset{i=1}{\sum }}P_{ij}\ln\left(P_{ij}\right),j$ =1, 2, …, m ( $k=\frac{1}{\ln\left(n\right)}>0$ , so $e_{j}\geq 0$ ).

Step 5: Calculate the redundancy of information entropy.

$d_{j}=1-e_{j}$ , j =1, 2, …, m

Step 6: Calculate the weights of each indicator.

$W_{j}=\frac{d_{j}}{\sum_{j=1}^{m}d_{j}},j$ =1, 2, …, m

Step 7: Calculate the comprehensive scores for each sample.

$s_{i}=\overset{m}{\underset{j=1}{\sum }}W_{j}P_{ij},i$ =1, 2,…, n
